# Genetic and Multi‐Omics Insights Into Monocyte Pantothenate‐Mediated Protection in Acute Respiratory Distress Syndrome

**DOI:** 10.1111/jcmm.70812

**Published:** 2025-10-08

**Authors:** Yang Wang, Hongyu Sun, Fengying Liang, Yanting Qian, Yuanyuan Wang, Mingdeng Wang, Yuansheng Lin

**Affiliations:** ^1^ Department of Intensive Care Unit, Suzhou Research Center of Medical School, Suzhou Hospital, Affiliated Hospital of Medical School Nanjing University Suzhou China; ^2^ Department of Respiratory Medicine, Suzhou Research Center of Medical School, Suzhou Hospital, Affiliated Hospital of Medical School Nanjing University Suzhou China

**Keywords:** ARDS, machine learning, Mendelian randomisation, metabolism, multi‐omics analysis

## Abstract

Acute respiratory distress syndrome (ARDS) is a severe condition with complex pathogenesis, and emerging evidence highlights the potential role of metabolic factors, though the exact mechanisms are not fully understood. In this study, we used Mendelian randomisation (MR) and multi‐omics approaches to investigate the causal relationship between plasma metabolites, immune cell profiles and ARDS risk. MR analysis of 1400 metabolites identified two causal metabolites linked to increased ARDS risk, primarily involved in pantothenate and CoA biosynthesis. Single‐cell RNA sequencing of ARDS samples revealed that monocytes exhibited the highest levels of pantothenate synthesis. Intercellular communication and pseudotime analysis suggested that the pantothenate synthesis pathway influenced monocyte differentiation and interactions with other cell types. Gene set enrichment analysis showed that monocytes with high pantothenate synthesis were significantly enriched in phagocytosis‐related pathways. Subsequent MR analysis demonstrated that CD33dim HLA DR+ CD11b+%CD33dim HLA DR+ were a risk factor against ARDS. Notably, monocytes with high pantothenate synthesis exhibited decreased expression of antigen presentation markers HLA‐DRB5, HLA‐DRB1 and HLA‐DRA, suggesting that the high pantothenate synthesis monocytes exhibit attenuated antigen presentation and enhanced phagocytic function. Moreover, we developed a diagnostic model using machine learning algorithms. Shapley Additive explanation (SHAP) was leveraged to evaluate the model performance, with CALM2 identified as the most influential feature across the CatBoost and XGBoost models. In summary, this study integrates genetic, multi‐omics and machine learning approaches to provide novel insights into the pathogenesis of ARDS and its potential therapeutic strategies targeting monocyte metabolism and function.

## 1 Introduction

Acute respiratory distress syndrome (ARDS) is a severe and life‐threatening condition characterised by rapid onset of widespread inflammation and increased permeability of the alveolar‐capillary barrier, leading to impaired gas exchange and respiratory failure [1, 2]. Despite advances in supportive care, including lung‐protective ventilation strategies and neuromuscular blockade, the mortality rate for ARDS remains high, ranging from 30% to 45% [3]. This persistent mortality burden highlights the critical need for a deeper understanding of the underlying pathophysiology to inform the development of more effective therapeutic interventions.

Emerging evidence suggests that metabolic factors may play a crucial role in the pathogenesis of ARDS, though the precise mechanisms remain incompletely understood [4, 5]. Alterations in cellular metabolism, including impaired energy production, oxidative stress and dysregulated signalling pathways, have been implicated in the initiation and progression of ARDS [6, 7]. Furthermore, metabolic disturbances, such as hyperglycemia, dyslipidaemia and mitochondrial dysfunction, have been associated with worse clinical outcomes in ARDS patients [8, 9, 10, 11]. However, the specific metabolic pathways and mediators involved in the development and resolution of ARDS remain poorly defined [12]. Continued research is necessary to elucidate this complex interplay between metabolism and the pathogenesis of ARDS, which may lead to the identification of novel therapeutic targets and the development of more personalised treatment strategies.

In this study, we aim to investigate the causal relationship between plasma metabolites, immune cell profiles and the risk of ARDS using Mendelian randomisation (MR) analysis. We integrate single‐cell sequencing and transcriptomic data to explore the potential mechanisms underlying these associations. Additionally, we employ machine learning approaches to evaluate the predictive performance of a comprehensive set of risk factors for ARDS development. By elucidating the causal pathways and underlying biological mechanisms linking these metabolic and immune factors to ARDS, our research is expected to provide important insights into the pathogenesis of this devastating syndrome.

## 2 Methods

### 2.1 Data Collection

Genome‐wide association study (GWAS) data for ARDS were obtained from the FinnGen consortium. The ARDS GWAS data set included 216,363 samples and 16,380,461 single nucleotide polymorphisms (SNPs) [13]. Additionally, we acquired GWAS summary statistics for 1400 blood metabolite levels from the GWAS Catalogue database (GCST90199621 to GCST90201020), including 1091 blood metabolites and 309 metabolite ratios [14]. GWAS data for 731 immune cell phenotypes, including 118 absolute cell counts, 389 surface antigen levels, 32 morphological features and 192 relative cell counts were obtained from the IEU Open GWAS project (ebi‐a‐GCST0001391 to ebi‐a‐GCST90002121) [15]. To further investigate the critical cell types associated with ARDS, we downloaded peripheral blood mononuclear cell (PBMC) scRNA‐seq data sets for ARDS (GSE180578) from the Gene Expression Omnibus (GEO), and the GSE32707 and GSE243068 provided microarray‐based transcriptomic profiles of whole blood samples from individuals with ARDS for machine learning [16, 17, 18].

### 2.2 MR Analysis and MetaboAnalyst Analysis

For the Mendelian randomisation (MR) analysis, we employed a *p* value threshold of < 1 × 10^−6^ to identify SNPs significantly associated with the metabolites and *p* < 5 × 10^−6^ for immune cells based on previous studies [19, 20]. Instrumental variables (IVs) were selected after clumping, using a linkage disequilibrium (LD) threshold of *r* ^2^ < 0.001 within a 10,000 kb distance and the European 1000 Genomes Project Phase 3 reference panel [21]. We then used PhenoScanner to remove IVs related to ARDS, which were considered confounding factors. Additionally, palindromic SNPs with a minor allele frequency (MAF) below 0.01 were excluded. The effectiveness of each IV was evaluated using the *F*‐statistic, and metabolites and immune cells with an *F*‐statistic less than 10 were removed. The inverse variance‐weighted (IVW) method was the main method used for our MR study, and other analyses included MR‐Egger, weighted median, weighted mode and simple mode as supplementary analyses [22]. Cochran's *Q* statistic was utilised to evaluate the heterogeneity of IVs, and MR‐Egger regression was employed to identify any horizontal pleiotropy. To ascertain the effect of specific SNPs on the result, we further conducted leave‐one‐out sensitivity analysis [23, 24]. Using the MetaboAnalyst 5.0 online tool (https://www.metaboanalyst.ca/), the names of the metabolites that had *p* values from the IVW method less than 0.05 were translated to KEGG IDs for pathway enrichment analysis [25].

### 2.3 ScRNA‐Seq Data Processing and Functional Analysis

The scRNA‐seq data were processed and analysed using the well‐established Seurat pipeline [26]. We first performed data normalisation using the LogNormalise function. Highly variable genes were then identified, and principal component analysis (PCA) was conducted on these genes. Cell clustering was carried out using the FindNeighbors and FindClusters functions, and uniform manifold approximation and projection (UMAP) dimensionality reduction was performed, with cell types manually annotated based on the SingleR package. The immune cell proportions of each sample were displayed using the scRNAtoolVis package. Immune cell metabolic activity was scored based on KEGG metabolic gene sets, followed by pseudotime analysis and cell–cell interaction analysis for each cell subtype [27, 28]. Transcription factor activity in immune cells was analysed using the DoRothEA package [29]. Differential gene expression analysis and gene set enrichment analysis (GSEA) were performed using the FindMarkers function and the clusterProfiler package respectively, in which GSEA gene sets were obtained from the ‘c2.cp.Reactome.v2023.2’ collection of the MSigDB database.

### 2.4 Bulk RNA‐Seq Data Processing and the LASSO Regression Algorithm

For the bulk transcriptome data GSE32707 and GSE235357, the sva package was employed to correct for batch effects between the two data sets, and the ComBat function was applied to further remove any residual batch effects. The expression values were then log2‐transformed for normalisation, and common genes between the two data sets were identified for subsequent analysis. To identify the key genes associated with ARDS, least absolute shrinkage and selection operator (LASSO) regression was performed on the preprocessed transcriptome data using the glmnet package. A 10‐fold cross‐validation was conducted to determine the optimal LASSO penalty parameter λ that minimised the cross‐validated error. Genes with nonzero coefficients in the LASSO model at the optimal λ were selected as the key feature genes. Moreover, the IOBR package was employed to quantify the association between the expression of the key feature genes and the infiltration levels of various immune cell types, including T cells, B cells, natural killer cells and myeloid cells, in the ARDS samples [30].

### 2.5 Machine Learning and SHAP Explanation

In parallel, we trained three advanced machine learning models, CatBoost, NGBoost and XGBoost to evaluate the identified key genes related to the disease [31]. Briefly, the data set was randomly split into training and test sets, with the training set comprising 80% of the samples and the test set comprising the remaining 20%. We trained the models on the training set and evaluated their performance on the test set using the area under the receiver operating characteristic (ROC) curve (AUC) metric. To further understand the contribution of each feature to the model predictions, we employed Shapley additive explanations (SHAP), in which SHAP values quantify the importance of each feature in the model outputs, enabling the identification of the most influential genes [32].

### 2.6 Statistical Method

The correlation between the variates was examined using the Spearman technique. The Kaplan–Meier test employed the log rank *p* value. A *p* value of less than 0.05 with two tails was deemed significant. The analysis was carried out utilising Python (3.11.8) or *R* (4.2.2).

## 3 Result

### 3.1 Metabolomic MR Analysis Identifies Pantothenate and CoA Biosynthesis Alterations Associated With ARDS

**FIGURE 1:**
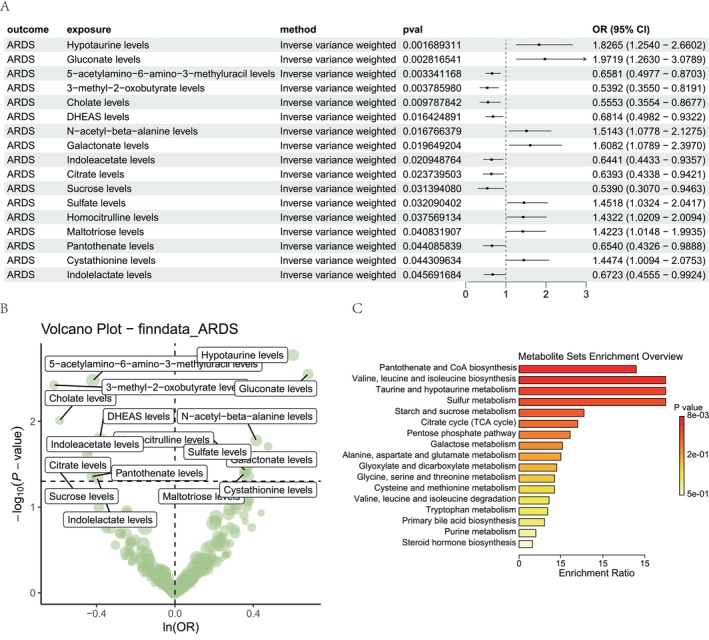
Mendelian randomisation analysis identify pantothenate and CoA biosynthesis pathways associated with ARDS. (A) Forest plot displaying the results of the Mendelian randomisation analysis, with metabolites significantly associated with ARDS risk shown. (B) Volcano plot of the Mendelian randomisation‐identified metabolites. (C) Bar plot showing the levels of specific metabolites driving the enrichment of the pantothenate and CoA biosynthesis pathway.

MR analysis was employed to identify metabolites with causal relationships to ARDS. From the metabolomic profile, 17 metabolites were found to be significantly associated with ARDS risk (Figure 1A,B). To gain further mechanistic insights, we performed metabolite set enrichment analysis on the MR‐identified metabolites. This revealed significant enrichment in pathways related to pantothenate and CoA biosynthesis as well as other amino acid and energy metabolism pathways (Figure 1C). The pantothenate and CoA biosynthesis pathway was the most significantly enriched, in which specific metabolites driving this enrichment included decreased levels of pantothenate (*p* = 0.044085839; OR [95% CI] = 0.6540 [0.4326–0.9888]), as well as the levels of the intermediates 5‐acetylamino‐6‐amino‐3‐methyluracil (*p* = 0.003341168; OR [95% CI] = 0.6581 [0.4977–0.8703]). These findings indicate dysregulation of pantothenate biosynthesis may be a protective response against ARDS.

### 3.2 Increased Pantothenate Synthesis in Monocytes of ARDS Patients

**FIGURE 2:**
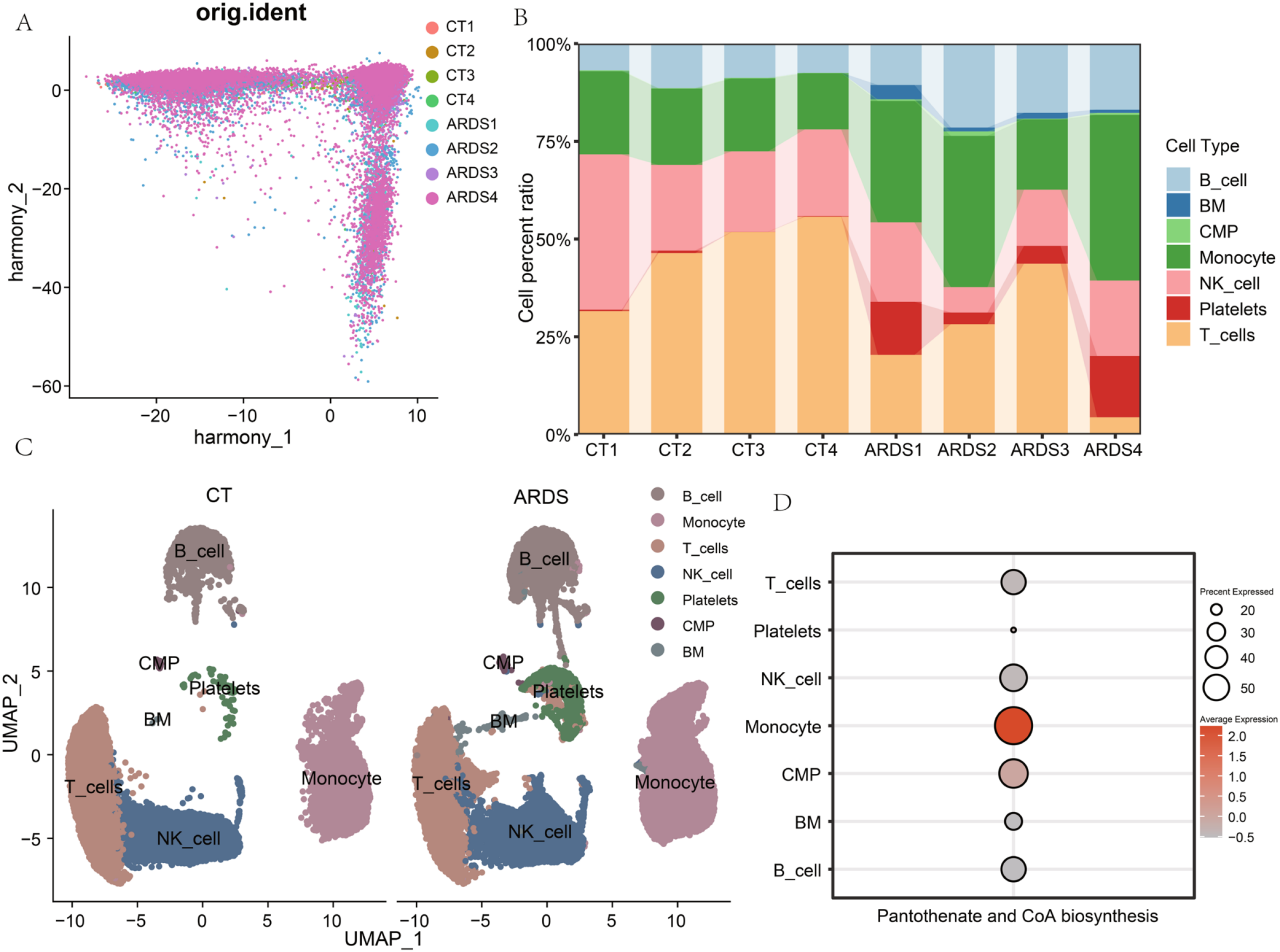
Single‐cell transcriptomic evidence for increased pantothenate synthesis in ARDS monocytes. (A) UMAP visualisation of single‐cell transcriptomic data showing harmonised clustering between control and ARDS samples. (B) Cell type composition plot from single‐cell data. (C) UMAP plot annotating the major cell types in ARDS and control samples. (D) Pantothenate synthesis scoring in single cells. The heatmap shows the expression levels of genes involved in the pantothenate and CoA biosynthesis pathway across different cell types.

Single‐cell transcriptomic data showed harmonised clustering of four ARDS patient samples and four control samples after quality control and normalisation (Figure 2A, Figure S1A–D). Further analysis of the cellular composition changes demonstrated a clear separation between the major cell types, and a significant increase in the relative abundance of monocytes in ARDS patients compared to controls (Figure 2B,C). Considering the pantothenate and CoA biosynthesis pathway was associated with ARDS, we performed gene set enrichment analysis on various cell types in ARDS samples. This analysis showed the monocytes exhibited a significant upregulation of the pantothenate and CoA biosynthesis pathway, compared to other major cell types (Figure 2D, Figure S1E). Pantothenate is a precursor for coenzyme A (CoA), a critical cofactor involved in numerous metabolic processes [33]. The elevated panacyl synthesis in ARDS monocytes suggests heightened demand for CoA, potentially reflecting altered cellular bioenergetics or signalling in these cells during the disease state.

### 3.3 Intercellular Communication Features and Single‐Cell Trajectory of Monocyte Subsets

**FIGURE 3:**
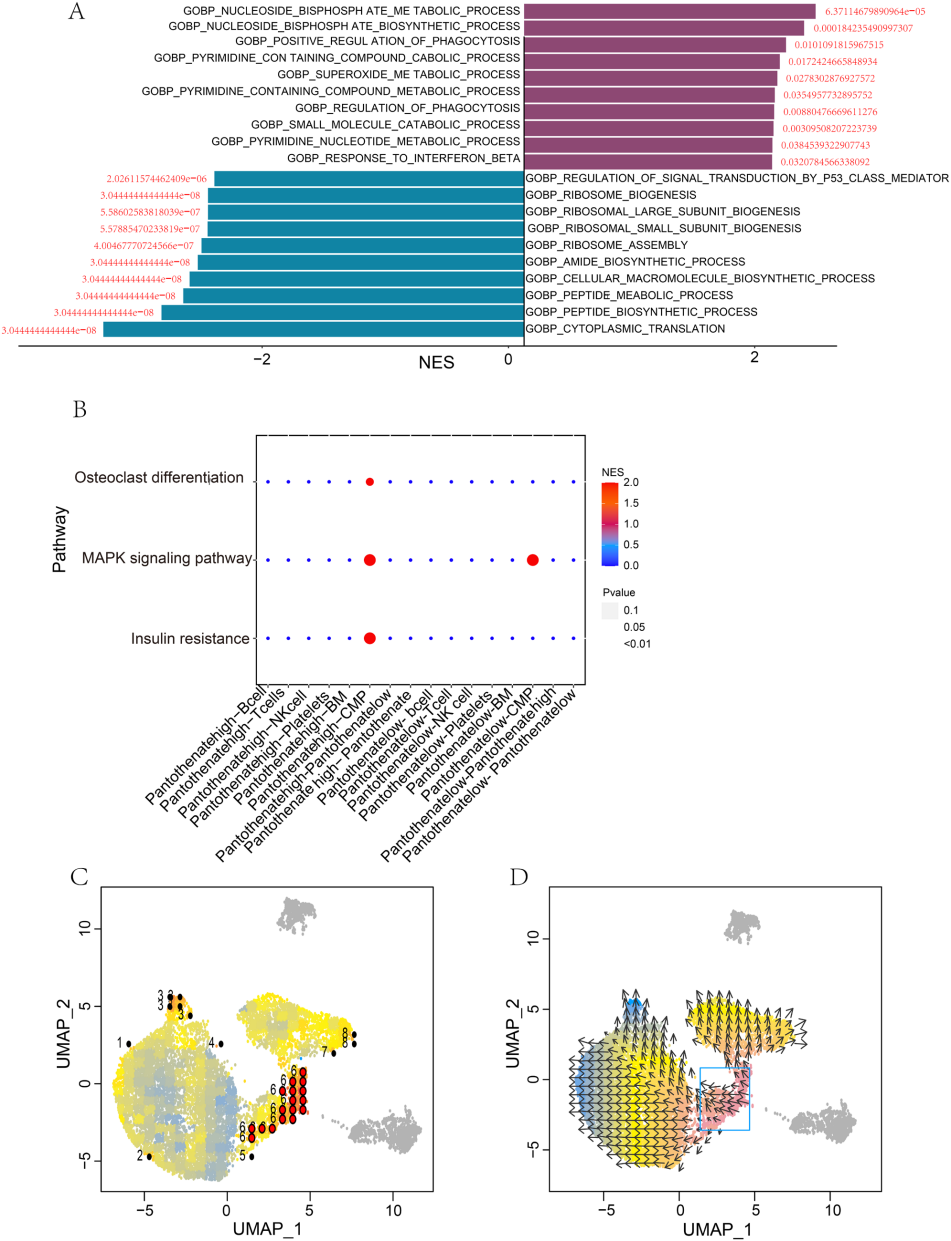
Pantothenate synthesis pathway shapes the development and intercellular communication of monocytes in ARDS. (A) Intercellular communication network of high vs. low pantothenate synthesis monocytes. (B) The VECTOR plot displays the inferred pseudotime trajectory, revealing a continuum of cellular states from early to late stages. (C) Cells at the start of the trajectory have the highest pantothenate synthesis activity, suggesting it may be lost during disease progression.

To further elucidate the dynamic states and intercellular communication patterns associated with monocyte subsets, we performed cell–cell interaction mapping and single‐cell trajectory analysis. We re‐clustered the monocyte population using the harmony algorithm and visualised the subpopulations on a UMAP plot (Figure S2A–C). We then calculated the normalised enrichment scores for the pantothenate and CoA biosynthesis pathways across the monocyte subsets, which revealed distinct expression patterns (Figure S2D). The monocytes were stratified into high and low pantothenate synthesis subsets based on the pathway scores. Interestingly, when examining the cell–cell communication networks, we found that the high pantothenate synthesis monocytes exhibited differential interactions with the common myeloid progenitor population in insulin resistance and osteoclast differentiation pathways (Figure 3A). Specifically, the high pantothenate synthesis monocytes showed enhanced signalling through TNF‐TNFRSF1A pathways, as evidenced by the enriched expression of relevant ligands and receptors in the cell–cell communication heatmap (Figure S2E). Furthermore, the pseudotime trajectory analysis provided insights into the potential developmental origins and dynamic states of monocyte subsets. These data suggest that the metabolic reprogramming toward increased pantothenate synthesis may coincide with distinct functional and signalling features that could shape the ARDS disease course.

### 3.4 The High Pantothenate Synthesis Monocytes Exhibited Attenuated Antigen Presentation and Enhanced Phagocytic Function

**FIGURE 4:**
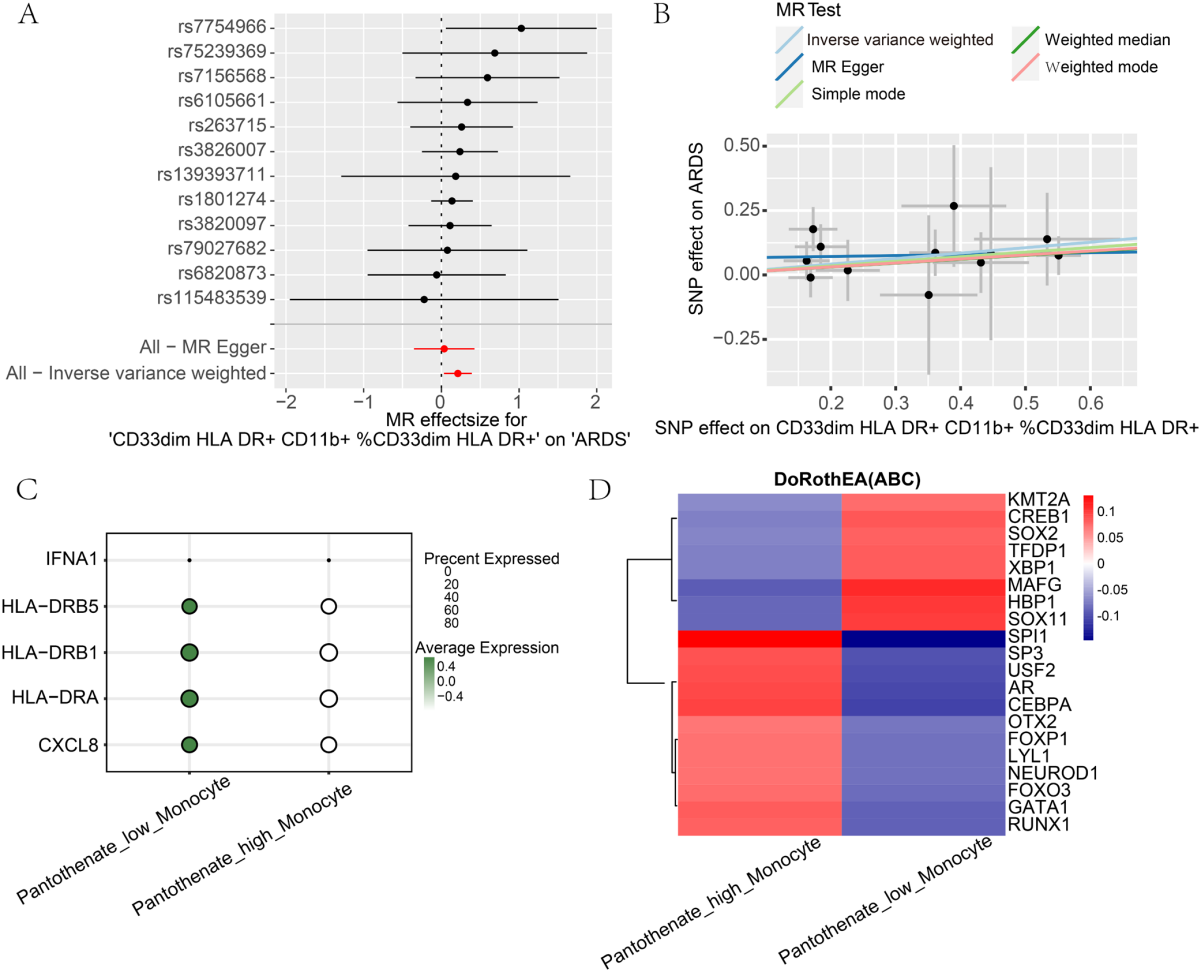
Functional enrichment of high vs. low pantothenate synthesis monocytes. (A) GO pathway analysis reveals monocytes with high pantothenate synthesis activity exhibit increased enrichment of phagocytosis, and other immune‐related pathways. (B) Mendelian randomisation further identified a causal association between the frequency of antigen‐presenting CD33dim HLA‐DR+ CD11b + monocytes and increased ARDS risk. (C) Immune‐related markers decreased expression in high pantothenate synthesis monocytes. (D) Transcription factor analysis between high versus low pantothenate synthesis monocytes.

Based on the previous observations of altered intercellular communication and pseudotemporal dynamics in high versus low pantothenate synthesis monocyte subsets, we further investigate the transcriptomic differences between these two populations mechanistically. Differential gene expression analysis revealed 159 differentially expressed genes (*p* < 0.05, log2|fold change| > 0.1) between the high and low pantothenate synthesis monocyte subsets. GO enrichment analysis of these differentially expressed genes indicated that the high pantothenate synthesis monocytes were significantly enriched for pathways related to phagocytosis (Figure 4A). This is noteworthy, as immune cells with heightened phagocytic capacity often exhibit reduced antigen presentation, reflecting an enhanced ability to clear pathogens without eliciting excessive inflammation [34]. To investigate this relationship, we employed MR to assess the causal effect of monocyte antigen presentation on ARDS risk. Interestingly, the level of CD33dim HLA DR+ CD11b + %CD33dim HLA DR+ (relative count) which is the monocyte subset representing the antigen‐presenting fraction, was identified as a risk factor for ARDS (Figure 4B). Consistent with this, we compared the expression levels of various immune‐related markers, such as HLA‐DRB5, HLA‐DRB1, HLA‐DRA and CXCL8, between the high and low pantothenate synthesis monocyte subsets (Figure 4C). The results showed that the high pantothenate synthesis monocytes exhibited decreased expression of the antigen presentation markers HLA‐DRB5, HLA‐DRB1 and HLA‐DRA. The expression of CXCL8, a molecule that recruits neutrophils also showed decreased level. The high pantothenate synthesis monocytes were shown to have a unique transcriptional signature, with upregulation of various transcription factors, according to an analysis of transcription factor activity (Figure 4D). All of these findings point to the possibility that enhanced monocyte pantothenate synthesis fosters a protective immune phenotype, which is marked by increased phagocytic activity and decreased antigen presentation. This phenotype may help to lessen the harmful inflammatory reactions linked to ARDS.

### 3.5 Building a Diagnostic Model With the SHAP Explanation Based on Bulk RNA‐Seq Data

**FIGURE 5:**
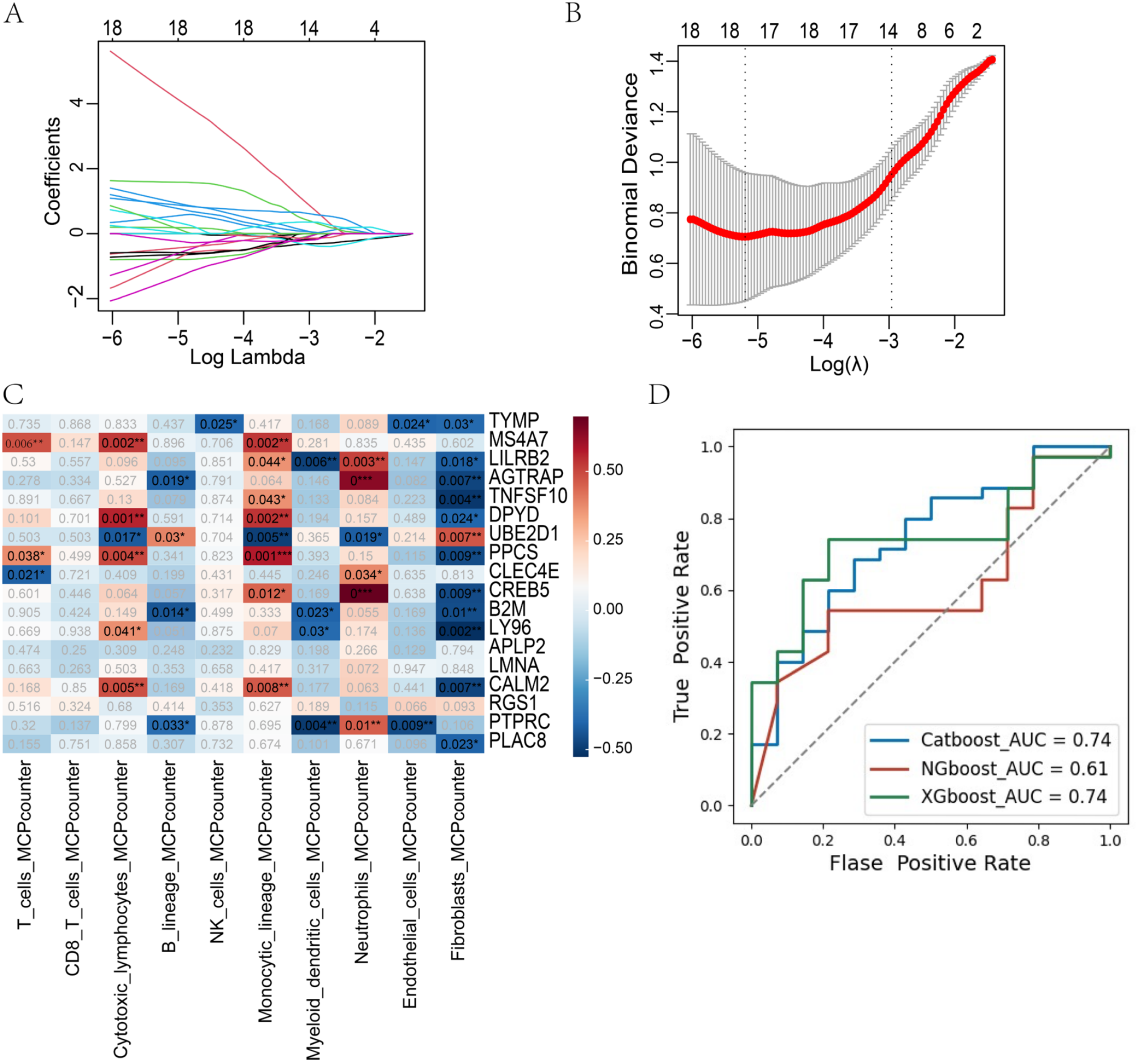
Building a diagnostic model for ARDS prediction. (A, B) LASSO regression of pantothenate synthesis genes in monocytes. (C) Correlation of pantothenate synthesis genes with immune cell populations. (D) Receiver operating characteristic curves of machine learning models for ARDS prediction.

To further investigate the clinical importance of monocytes with high pantothenate synthesis, we developed a diagnostic model based on PBMC bulk RNA sequencing data from ARDS patients. The data set GSE32707 was used as the training set and GSE243068 as the validation set. Through rigorous LASSO regression analysis, we identified 18 core genes that exhibited robust association with ARDS pathogenesis among 159 differentially expressed genes, and the 18 key genes selected by the LASSO model were then used as the input features for the subsequent machine learning model development and validation (Figure 5A,B). We utilised the IOBR package to analyse the relationship between the expression of these 18 core genes and immune cell infiltration. The results showed that the expression levels of these core genes were significantly correlated with the abundances of neutrophils, monocytes and cytotoxic T cells (Figure 5C).

**FIGURE 6:**
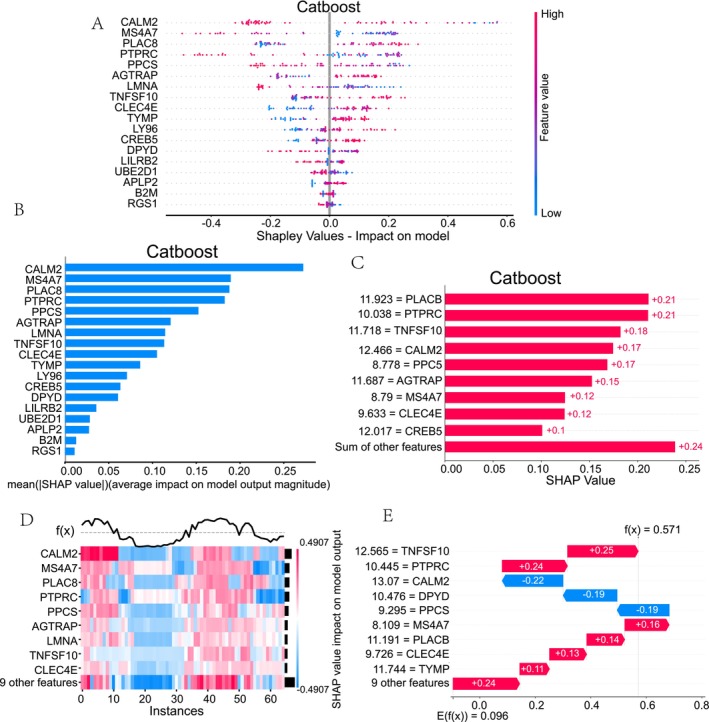
The hub markers detection based on CatBoost and SHAP model. (A) Summary plot of CatBoost model. (B, C) The SHAP value of each gene in CatBoost model. (D, E) Bar plot and waterfall plot for individual sample on CatBoost model.

**FIGURE 7:**
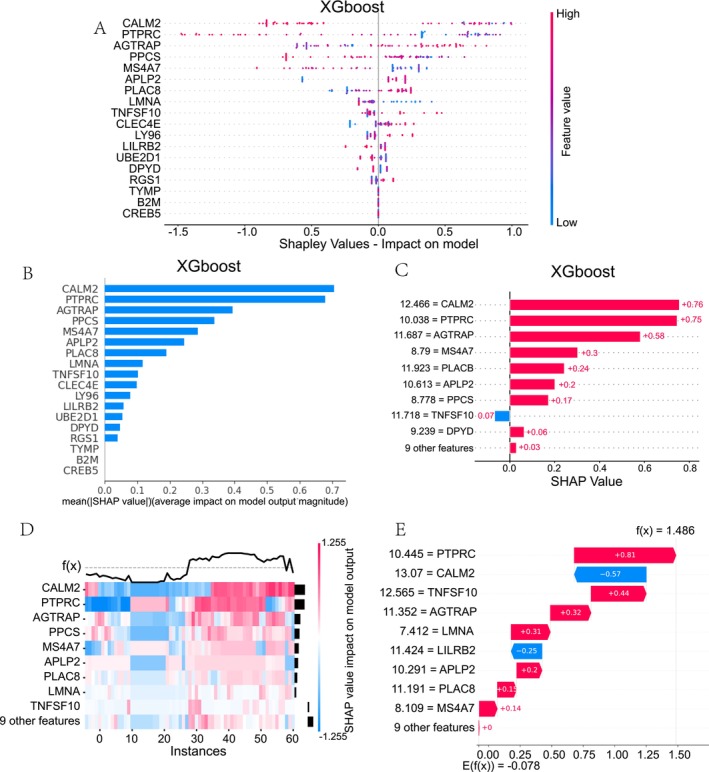
The hub markers detection based on XGBoost and SHAP model. (A) Summary plot of XGBoost model. (B, C) The SHAP value of each gene in XGBoost model. (D, E) Bar plot and waterfall plot for individual sample on XGBoost model.

Furthermore, we used three machine learning algorithms, CatBoost, NGBoost and XGBoost, to develop the diagnostic model separately. The AUC metric was used to evaluate the models’ performance on the validation set. We discovered that the CatBoost and XGBoost models had better performance with AUC values greater than 0.7, indicating the strong potential of the selected 18 gene signature for accurate ARDS diagnosis (Figure 5D). Therefore, we further leveraged SHAP, a model‐agnostic approach to gain deeper insights into the specific contributions of individual genes within the predictive models (Figures 6A, 7A). The SHAP value plot and heatmap revealed that CALM2, exhibiting the largest SHAP values, was the most influential feature across both CatBoost and XGBoost models. This finding suggests that CALM2 is a crucial predictor that significantly contributes to the models’ discriminative power (Figures 6B,C and 7B,C). The SHAP analysis conducted on the individual sample not only confirmed the key drivers identified at the global model level but also enabled the exploration of patient‐specific variability (Figures 6D,E and 7D,E). For example, the analysis on individual sample indicated that higher values of TNFSF10 and PTPRC were associated with increased model predictions, whereas elevated levels of CALM2 and CLEC4E correspond to lower predictions (Figure 6E). Of particular note, CALM2 has been previously implicated in pathogenic mechanisms underlying ARDS development.

## 4 Discussion

ARDS, characterised by rapid onset of widespread inflammation and increased permeability of the alveolar‐capillary barrier, leading to impaired gas exchange and respiratory failure, is a life‐threatening disease with a high mortality rate [35]. ARDS is driven by a dysregulated immune response, with excessive activation of inflammatory pathways and disruption of normal lung homeostasis [36]. Metabolic factors have emerged as important contributors to the development and progression of ARDS, though the precise mechanisms remain incompletely elucidated [5]. In this study, we employed MR and multi‐omics analysis to reveal the protective mechanism of monocyte pantothenate synthesis against ARDS, providing important insights into the complex interplay between metabolism and immune function in this devastating condition.

Pantothenate, also known as vitamin B5, is an essential nutrient required for the biosynthesis of CoA, which is a critical cofactor involved in numerous metabolic pathways [37]. Previous studies have highlighted the importance of pantothenate and CoA metabolism in regulating inflammatory processes and immune cell function [38, 39]. Our results align with three foundational biological theories: 1. Metabolic Reprogramming Theory: Elevated pantothenate synthesis fuels CoA production, redirecting monocyte metabolism toward phagocytosis (energy‐intensive) and away from antigen presentation—a functional trade‐off that dampens immunopathology [40]. 2. Calcium‐CALM2 Axis: Dysregulated CALM2 (Figures 6, 7) signifies pantothenate‐mediated disruption of Ca^2+^ signalling, which normally potentiates NF‐κB‐driven inflammation and barrier damage [41]. 3. Phagocytic Priority Principle: The inverse correlation between phagocytosis (GO:0006909) and HLA‐II expression (Figure 4C) reflects an evolutionary adaptation to clear pathogens without excessive T‐cell activation [42]. Collectively, these mechanisms explain how pantothenate synthesis reprograms monocytes toward a protective phenotype in ARDS. Our findings suggest that increased pantothenate synthesis in monocytes may confer protection against the development of ARDS through its modulating effects on cellular metabolism and immune responses.

Monocytes are key players in the pathogenesis of ARDS, as they are responsible for initiating and propagating the inflammatory cascade that leads to alveolar‐capillary barrier disruption and impaired gas exchange [43, 44]. The dysregulation of pantothenate metabolism in monocytes/macrophages can have far‐reaching effects on the broader immune microenvironment [45, 46]. Reduced pantothenate synthesis leads to decreased intracellular CoA levels, which impair mitochondrial function and energy metabolism in these immune cells [47]. This metabolic reprogramming can result in increased oxidative stress and the production of pro‐inflammatory mediators [47]. Furthermore, CoA‐dependent protein acetylation regulates key signalling pathways involved in the inflammatory response, and its disruption can lead to aberrant cytokine production by monocytes/macrophages [48]. Importantly, pantothenate metabolism also influences the phagocytic and clearance functions of these cells, and impaired clearance of apoptotic debris can contribute to the perpetuation of inflammation and autoimmunity [45]. Collectively, the metabolic and functional changes in monocytes/macrophages driven by altered pantothenate synthesis have the potential to shape the overall inflammatory milieu, with implications for the pathogenesis of diverse immune‐mediated disorders. Elucidating these intricate relationships provides valuable insights that may guide the development of novel therapeutic strategies targeting the metabolic underpinnings of inflammatory conditions.

Through the application of various machine‐learning techniques, our research has identified the CALM2 gene as a critical differentially expressed gene associated with pantothenate synthesis in monocytes, and CALM2 holds significant implications for the diagnosis and understanding of ARDS. CALM2 encodes the calmodulin 2 protein, which plays a crucial role in calcium signalling and a wide range of cellular processes [49]. Interestingly, calmodulin has been previously implicated in the pathogenesis of ARDS, as it is involved in regulating inflammatory responses, endothelial barrier function and alveolar epithelial cell integrity [50]. The dysregulation of CALM2 expression, as a result of altered pantothenate metabolism in monocytes, may serve as a valuable diagnostic biomarker for ARDS. Aberrant CALM2 levels could reflect the underlying metabolic and signalling perturbations in these key immune cells, which contribute to the development and progression of ARDS.

## 5 Limitations of the Study

While our integrative multi‐omics approach provides novel insights into the role of pantothenate and CoA biosynthesis in ARDS, we acknowledge the need for experimental validation to confirm the causal relationships and mechanistic insights suggested by our findings. Future studies will include in vitro and in vivo experiments to validate the functional roles of the identified metabolites, genes and monocyte subsets in ARDS pathogenesis. Additionally, expanding our single‐cell and bulk RNA‐seq analyses to larger, more diverse cohorts will enhance the generalisability of our findings and refine the diagnostic model. Addressing these limitations will be critical for translating our results into clinically actionable strategies. Our study primarily utilised European‐ancestry cohorts, which may limit generalisability to other populations. Additionally, experimental validation of the causal role of pantothenate synthesis in monocytes is needed.

Despite these limitations, the consistency of our MR results across geographically distinct data sets—including replicated protection by pantothenate in Biobank Japan—suggests a conserved biological role of pantothenate‐mediated immunity in ARDS. Future studies in admixed populations are warranted to validate these findings and elucidate potential ancestry‐specific modifiers of pantothenate metabolism.

## 6 Conclusion

Our integrative Mendelian randomisation and multi‐omics analysis has uncovered the protective role of monocyte pantothenate synthesis in ARDS pathogenesis. These results highlight the importance of metabolic factors, particularly those related to pantothenate and CoA metabolism, in regulating the immune response and susceptibility to this life‐threatening condition. Further research is warranted to elucidate the precise molecular mechanisms by which pantothenate and pantothenate synthesis –related pathways influence monocyte function and ARDS development, which may inform the development of novel therapeutic strategies targeting metabolic pathways in the management of this devastating syndrome.

## Author Contributions

**Yang Wang:** resources (equal), validation (equal). **Hongyu Sun:** validation (equal), writing – original draft (equal). **Fengying Liang:** funding acquisition (equal), supervision (equal). **Yanting Qian:** resources (equal), writing – original draft (equal). **Yuanyuan Wang:** conceptualization (equal), data curation (equal). **Mingdeng Wang:** validation (equal), visualization (equal). **Yuansheng Lin:** investigation (equal), project administration (equal).

## Ethics Statement

The research involving human subjects was reviewed and approved by the Ethics Committee of Suzhou Science and Technology City Hospital.

## Conflicts of Interest

The authors declare no conflicts of interest.

## Supporting information


**Figure S1.** Data preprocessing and dimensionality reduction. (A, B) Quality control steps, including filtering out low‐quality cells and genes with low expression, are applied to the single‐cell data set. (C) Principal component analysis (PCA) is used to reduce the high‐dimensional single‐cell data into a lower dimensional space. (D) The batch effects were removed using the Harmony package.
**Figure S2.** Monocyte subpopulation analysis. (A–C) The monocyte subpopulation is visualised after dimensionality reduction and batch effect removal, revealing distinct subpopulations. (D) Monocyte subsets are further characterised by their pantothenate synthesis pathway activity scores. (E) Heatmap depicted the intercellular communication network between monocyte subsets and other immune cells.

## Data Availability

The datasets generated and/or analyzed during the current study are made available to the corresponding author upon reasonable request.
