# Supplementary material for: Genetic and Multi‐Omics Insights Into Monocyte Pantothenate‐Mediated Protection in Acute Respiratory Distress Syndrome
Source: J Cell Mol Med. 2025 Oct 8;29(19):e70812. doi: 10.1111/jcmm.70812 (PMC12505201; doi:10.1111/jcmm.70812)

Supplementary figure 1. Data preprocessing and dimensionality reduction.

(A, B) Quality control steps, including filtering out low-quality cells and genes with low expression, are applied to the single-cell dataset.

(C) Principal component analysis (PCA) is used to reduce the high-dimensional single-cell data into a lower dimensional space.

(D) The batch effects were removed using the Harmony package.


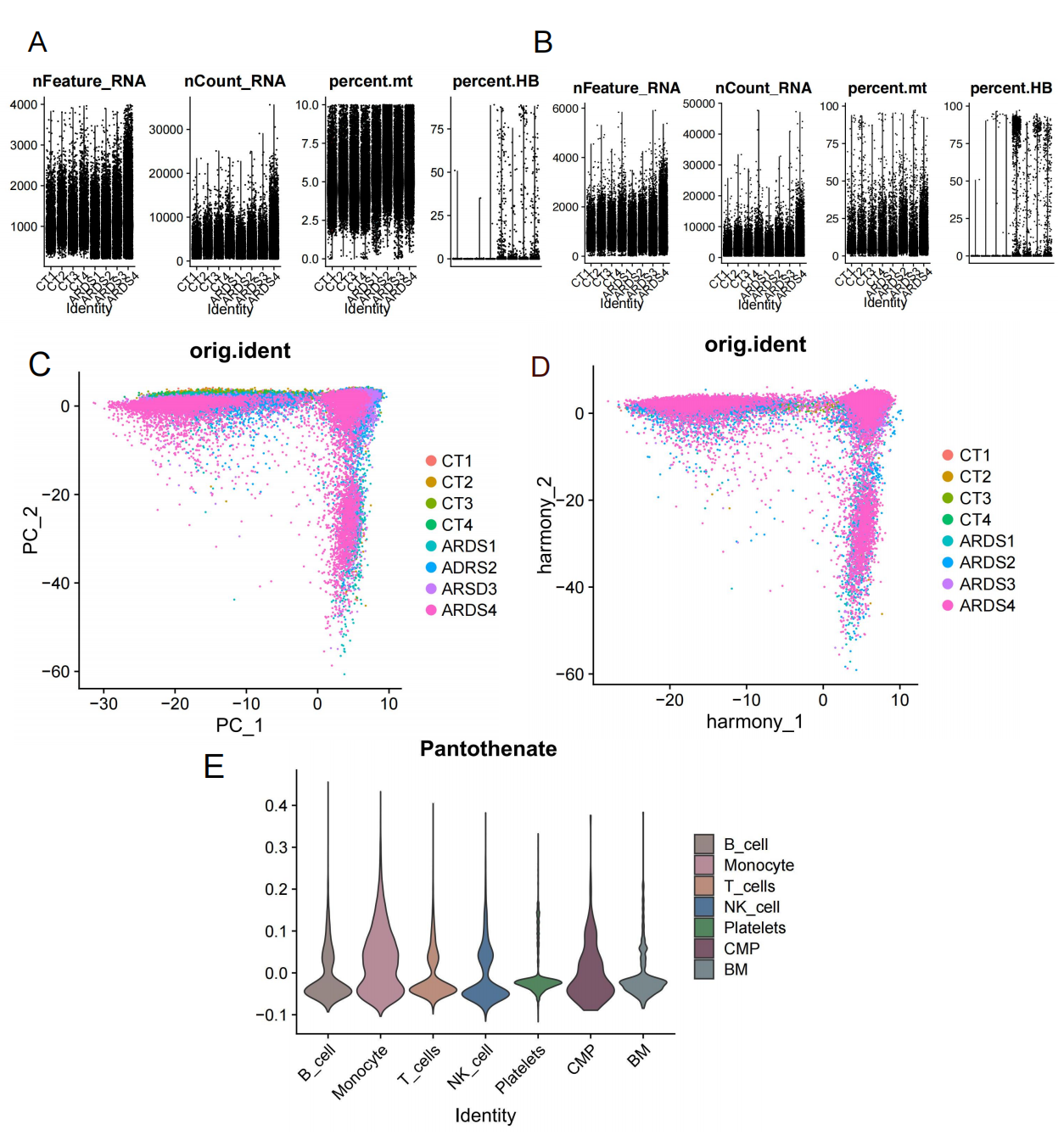


Supplementary figure 2. Monocyte subpopulation analysis.

(A-C) The monocyte subpopulation is visualized after dimensionality reduction and batch effect removal, revealing distinct subpopulations.

(D) Monocyte subsets are further characterized by their pantothenate synthesis pathway activity scores.

(E) Heatmap depicted the intercellular communication network between monocyte subsets and other immune cells.


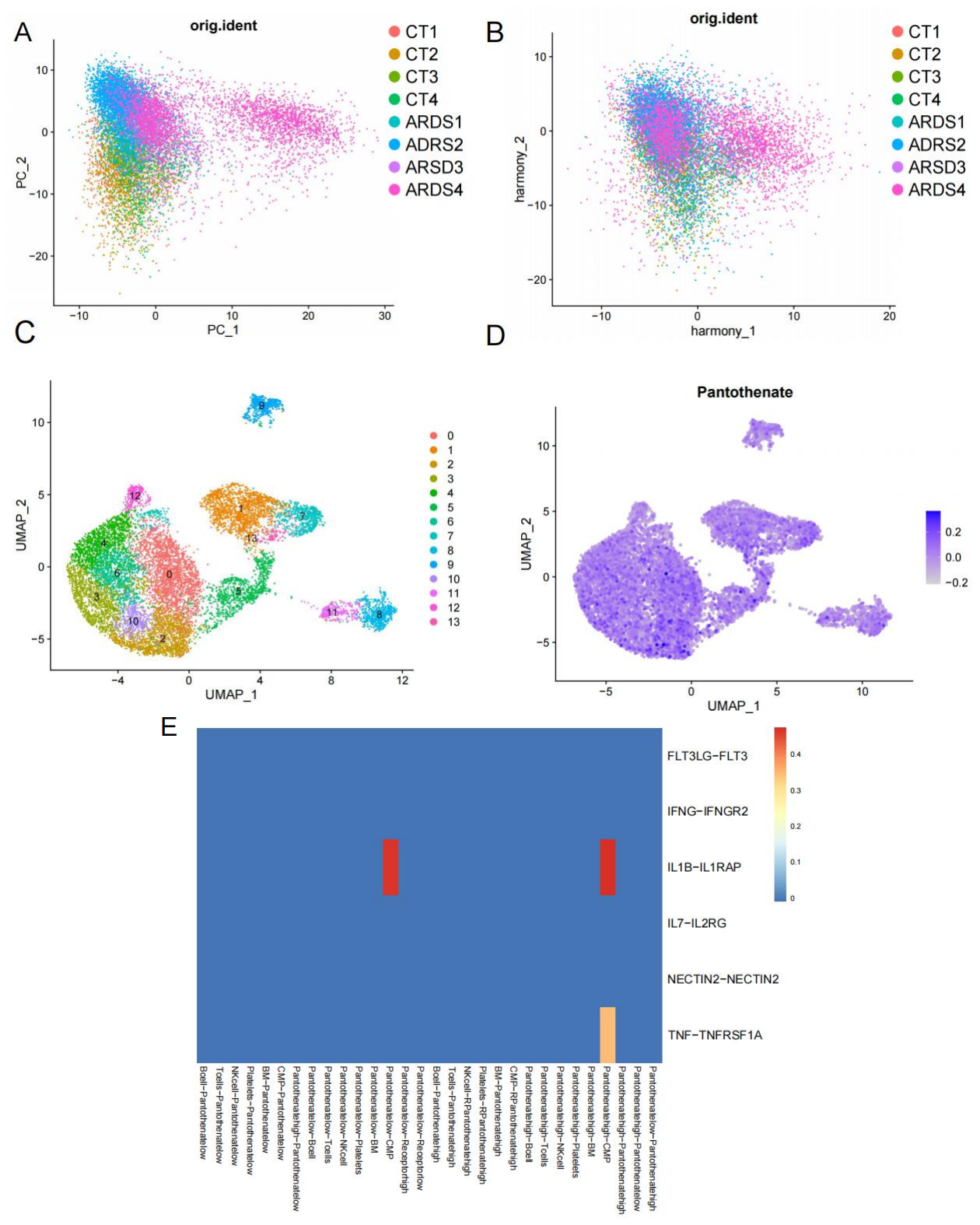

Supplement: Supplementary file 1 — Figure S1. Data preprocessing and dimensionality reduction. (A, B) Quality control steps, including filtering out low‐quality cells and genes with low expression, are applied to the single‐cell data set. (C) Principal component analysis (PCA) is used to reduce the high‐dimensional single‐cell data into a lower dimensional space. (D) The batch effects were removed using the Harmony package. Figure S2. Monocyte subpopulation analysis. (A–C) The monocyte subpopulation is visualised after dimensionality reduction and batch effect removal, revealing distinct subpopulations. (D) Monocyte subsets are further characterised by their pantothenate synthesis pathway activity scores. (E) Heatmap depicted the intercellular communication network between monocyte subsets and other immune cells. [file JCMM-29-e70812-s001.docx]
